# Resolving cognitive heterogeneity in white matter hyperintensities through integrated analyses of microbiome, metabolome, and brain glymphatic function

**DOI:** 10.1002/alz.71201

**Published:** 2026-02-17

**Authors:** Xiaotao Xu, Xia Zhou, Mengwei Zhang, Jingjing Fang, Zhiqing Yan, Xiaoqun Zhu, Yongqiang Yu, Jiajia Zhu

**Affiliations:** ^1^ Department of Radiology The First Affiliated Hospital of Anhui Medical University Hefei China; ^2^ Research Center of Clinical Medical Imaging, Anhui Province Hefei China; ^3^ Anhui Provincial Key Laboratory for Brain Bank Construction and Resource Utilization Hefei China; ^4^ Department of Neurology The First Affiliated Hospital of Anhui Medical University Hefei China

**Keywords:** brain glymphatic function, cognitive impairment, gut microbiome, plasma metabolome, white matter hyperintensities

## Abstract

**INTRODUCTION:**

Individuals with similar white matter hyperintensities (WMH) burden show heterogeneous cognitive outcomes, yet the biological mechanisms underlying this variability remain incompletely understood.

**METHODS:**

We integrated 16S rDNA sequencing, untargeted metabolomics, and multi‐modal magnetic resonance imaging (MRI) to comprehensively characterize gut microbiome, plasma metabolome, and brain glymphatic function in 56 healthy controls, 40 WMH with normal cognition (WMH‐NC), and 49 WMH with cognitive impairment (WMH‐CI).

**RESULTS:**

Group comparisons revealed differences in six bacterial genera, three plasma metabolites, and five glymphatic markers across three groups, with *Acetivibrio*, 1,5‐naphthalenediamine, beta‐uridine, free water fraction within the white matter, and index of diffusivity along the perivascular spaces (ALPS index) showing differences between WMH‐CI and WMH‐NC. Correlation and mediation analyses demonstrated associations between microbiota and cognition, mediated by tetradecyldiethanolamine and ALPS index.

**DISCUSSION:**

These findings provide preliminary insights into plausible microbiota‐metabolites‐glymphatic function‐cognition associations in WMH, potentially informing more targeted interventions for vascular cognitive impairment.

## Highlights

- Multi‐omics study on cognitive heterogeneity in white matter hyperintensities (WMH)
- Microbiota, metabolites, and glymphatic markers linked to cognitive impairment in WMH
- Link between microbiota and cognition mediated by metabolite and glymphatic marker
- Preliminary insight into a more targeted intervention for vascular cognitive impairment

## 1 BACKGROUND

White matter hyperintensities (WMH) are areas of high signal intensity in the periventricular and deep cerebral white matter, typically visible on T2‐weighted or T2 fluid‐attenuated inversion recovery (FLAIR) images. WMH are almost ubiquitous neuroimaging findings in the elderly population^1^ and represent the earliest and most prevalent brain changes in cerebral small vessel disease (CSVD).^2^ Crucially, WMH are associated with an increased risk of cognitive dysfunction and constitute a leading cause of vascular cognitive impairment (VCI).^3^ However, in clinical practice, a puzzling phenomenon is often observed: some individuals with high WMH burden experience significant cognitive impairment (WMH‐CI), while others maintain relatively normal cognition (WMH‐NC). This divergence, that is, common WMH pathology but distinct cognitive trajectories, makes the cognition‐based subgrouping a particularly appropriate paradigm to dissect vulnerability (underlying WMH‐CI) and resilience (supporting WMH‐NC) to cognitive decline. Identifying the biological factors driving this variability is critical to unraveling modulatory mechanisms of VCI, thereby potentially providing a critical window for early interventions to prevent or delay the onset of VCI.

The glymphatic system is a brain‐wide perivascular network that clears metabolic waste through cerebrospinal fluid (CSF) and interstitial fluid (ISF) exchange, primarily mediated by astrocytes' aquaporin‐4 channels that drive CSF influx and clearance along perivenous routes.^4^ This process can be evaluated in vivo using some neuroimaging proxies.^5^, ^6^ Among these, we focused on five noninvasive magnetic resonance imaging (MRI)‐derived markers that delineate important aspects of the glymphatic pathway and have demonstrated relevance to cognition:^7^, ^8^, ^9^ choroid plexus volume (CPV) reflects CSF production^10^; perivascular spaces (PVS) serve as a proxy for CSF influx; free water (FW) estimates the efficiency of CSF‐ISF exchange; index of diffusivity along the perivascular spaces (ALPS index) serves as a proxy for ISF efflux^8^; and coupling between blood oxygen level‐dependent and cerebrospinal fluid signals (BOLD‐CSF coupling) reflects overall glymphatic system‐mediated waste clearance.^11^ Moreover, previous studies have demonstrated that larger CPV,^12^ higher PVS counts.^13^ higher FW,^14^ lower ALPS index,^15^, ^16^ and weaker BOLD‐CSF coupling strength^17^ are related to radiologic severity and CI in CSVD. These findings highlight the prospect of glymphatic dysfunction as a potentially mechanistic account for cognitive heterogeneity in WMH.

The gut microbiota cooperate with their hosts to impact brain development and function through dynamic bidirectional communication along the microbiota‐gut‐brain axis.^18^ Gut microbiota dysbiosis may affect the hosts’ behavior, cognition, and emotion, potentially contributing to brain disorders.^19^ Recently, growing research has begun to elucidate the role of the gut microbiota in CSVD.^20^, ^21^ For example, Cai et al. found that patients with arteriosclerotic CSVD exhibited distinct gut microbiota compositions compared to healthy controls (HC), which were linked to WMH.^20^ Notably, the bloodstream, transporting microbial metabolites, may bridge communication between the gut microbiota and the brain.^22^ Indeed, multiple circulating metabolites are associated with WMH in middle‐aged and older adults.^23^ Although our prior work established a plausible gut microbiome‐metabolome‐brain‐behavior pathway in CSVD patients with WMH,^21^ the fairly modest sample size and the lack of glymphatic markers limit subgroup analyses and interpretation from the perspective of the brain's intrinsic clearance system.

**FIGURE 1 alz71201-fig-0001:**
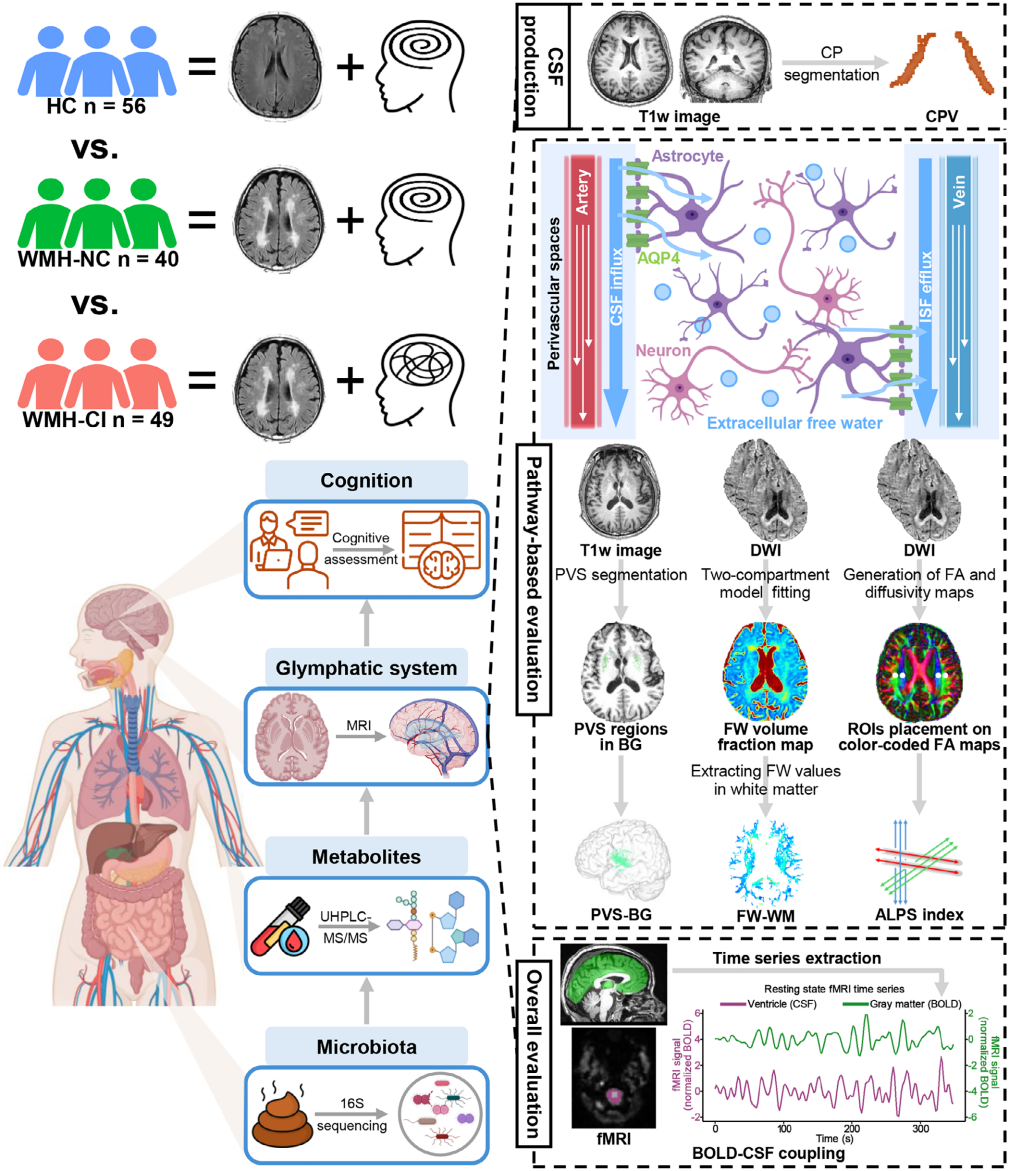
Research design and analytical procedure. We collected multi‑omics data from 56 HC, 40 WMH‐NC, and 49 WMH‐CI. 16S rDNA amplicon sequencing and untargeted metabolomics were used to quantify gut microbiota and plasma metabolites. Structural, diffusion, and resting‐state functional MRI data were utilized to calculate a range of glymphatic function markers to provide a comprehensive evaluation of the glymphatic system. Group differences in these multi‐omics measures were initially examined, followed by performance of correlation and mediation analyses with the aim of establishing a microbiota → metabolites → glymphatic system → cognition pathway. ALPS index, index of diffusivity along the perivascular spaces; AQP4, aquaporin‐4; BG, basal ganglia; BOLD, blood oxygen level‐dependent; BOLD‐CSF coupling, coupling between blood oxygen level‐dependent and cerebrospinal fluid signals; CPV, choroid plexus volume; CSF, cerebrospinal fluid; DWI, diffusion‐weighted imaging; FA, fractional anisotropy; fMRI, functional magnetic resonance imaging; FW, free water; FW‐WM, free water fraction within the white matter; HC, healthy controls; ISF, interstitial fluid; MRI, magnetic resonance imaging; PVS, perivascular spaces; PVS‐BG, perivascular spaces in the basal ganglia; ROIs, regions of interest; T1w, T1‐weighted; UHPLC‐MS/MS, ultrahigh performance liquid chromatography coupled with tandem mass spectrometry; WMH‐CI, white matter hyperintensities with cognitive impairment; WMH‐NC, white matter hyperintensities with normal cognition. Created in BioRender. xu, x. (2026) https://BioRender.com/kfbpx12.

In this study, we collected multi‑omics data from 56 HC, 40 WMH‐NC, and 49 WMH‐CI. 16S rDNA amplicon sequencing and untargeted metabolomics were used to quantify gut microbiota and plasma metabolites. Structural, diffusion, and resting‐state functional MRI data were utilized to calculate a range of glymphatic function markers to provide a comprehensive evaluation of the glymphatic system. Group differences in these multi‐omics measures were initially examined, followed by performance of correlation and mediation analyses with the aim of establishing a microbiota → metabolites → glymphatic system → cognition pathway (Figure 1).

## 2 METHODS

### 2.1 Participants

All participants (Chinese Han origin, right‐handed, aged 50–85 years) were recruited from The First Affiliated Hospital of Anhui Medical University between August 2018 and March 2022. Moderate‐severe WMH were defined as the sum of the periventricular WMH Fazekas score and deep WMH Fazekas score ≥3 on FLAIR images.^24^ Cognitive impairment was defined based on the Montreal Cognitive Assessment (MoCA) scores, with cutoffs determined according to educational levels of Chinese population: illiterate individuals ≤13, individuals with 1–6 years of education ≤19, individuals with 7 or more years of education ≤ 24.^25^ The exclusion criteria were as follows: (i) diagnosis of degenerative diseases of central nervous system, such as Alzheimer's disease (AD), Parkinson's disease (PD) and other nonvascular causes of brain injury‐induced dementia; (ii) brain tumors or other systemic malignancies; (iii) a history of traumatic brain injury or craniocerebral surgery; (iv) a history of ischemic stroke with a diameter of > 20 mm or cardiogenic cerebral infarction; (v) neuropsychological disorders; (vi) dysfunction of liver, kidney, heart, lung or other vital organs; and (vii) MRI scan contraindications. This study was approved by the Institutional Ethics Committee of The First Affiliated Hospital of Anhui Medical University (20200094) and was conducted following the Declaration of Helsinki. Written informed consent was obtained from all participants after a full explanation of the procedure.

**RESEARCH IN CONTEXT**

1. **Systematic review**: The authors reviewed literature using PubMed and Web of Science, examining gut microbiome, metabolome, and brain glymphatic dysfunction in white matter hyperintensities (WMH). No prior research has integrated multi‐omics approaches with comprehensive glymphatic markers to parse cognitive heterogeneity in WMH.
2. **Interpretation**: Our findings establish two potential pathways linking gut microbiota to cognition in WMH: microbiota → metabolites → glymphatic function → cognition, and microbiota → metabolites → cognition. Specifically, *Acetivibrio* depletion and *Staphylococcus* enrichment are associated with cognitive impairment, mediated by tetradecyldiethanolamine and index of diffusivity along the perivascular spaces, providing preliminary insights into plausible microbiota‐metabolites‐glymphatic function‐cognition associations in WMH.
3. **Future directions**: Critical questions include: (a) clinical trials examining whether interventions targeting aspects of the identified pathways can preserve cognition in WMH and prevent vascular cognitive impairment; (b) longitudinal and mechanistic research disentangling the causality of the multi‐omics associations.

### 2.2 Collection of demographic, clinical, and cognitive data

Demographic[Fig alz71201-fig-0001] and clinical data, including age, sex, years of education, and vascular risk factors, were collected through structured interviews and clinical assessments. Vascular risk factors were defined as follows: hypertension: systolic blood pressure ≥140 mmHg, or diastolic blood pressure ≥ 90 mmHg, or current use of antihypertensive medication; diabetes: fasting plasma glucose ≥ 7.0 mmol/L or current use of antidiabetic medication; dyslipidemia: total cholesterol ≥ 6.2 mmol/L, or triglycerides ≥ 2.3 mmol/L, or low‐density lipoprotein cholesterol ≥ 4.1 mmol/L, or high‐density lipoprotein cholesterol < 1.0 mmol/L, or current use of lipid‐lowering medication; smoking: smoking more than 10 cigarettes per day for more than 10 years. Cognitive assessment of all participants was performed by two trained neuropsychological technicians within 1 week of MRI examination. General cognition was evaluated by the MoCA and Mini‐Mental State Examination (MMSE). Moreover, episodic memory function was assessed using the 12‐word list from the Auditory Verbal Learning Test (AVLT). Attention and working memory were evaluated using the Forward Digit Span (FDS) and Backward Digit Span (BDS) from the Wechsler Adult Intelligence Scale‐Revised, respectively.

### 2.3 Collection of fecal and plasma samples

Fecal and blood samples were collected within 1 day before or after the MRI examination. Fecal samples were aliquoted into sterilized tubes using a scoop and immediately stored at −80°C. A 2 mL sample of fasting elbow vein blood was collected between 6:00 a.m. and 8:00 a.m. using Vacutainer tubes containing dipotassium ethylenediaminetetraacetic acid (EDTA‐K2), followed by immediate centrifugation at 3000 rpm for 10 min. The resulting plasma samples were then stored at −80°C.

### 2.4 Gut microbiomics analysis

The gut microbiome was quantified using 16S rDNA amplicon sequencing. Specifically, total genomic DNA from fecal samples was extracted using the cetyltrimethylammonium bromide (CTAB) method. DNA concentration and purity were monitored on 1% agarose gels. To construct a polymerase chain reaction (PCR) ‐based 16S rDNA amplicon library for sequencing, PCR enrichment of the V4 hypervariable region of 16S rDNA was performed with forward primer 515F (5′‐GTGCCAGCMGCCGCGGTAA‐3′) and reverse primer 806R (5′‐GGACTACHVGGGTWTCTAAT‐3′). Sequencing libraries were generated using TruSeq DNA PCR‐Free Sample Preparation Kit (Illumina, USA) following the manufacturer's recommendations, and index codes were added. Library quality was assessed on the Qubit@2.0 Fluorometer (Thermo Scientific) and Agilent Bioanalyzer 2100 system. At last, the library was sequenced on an Illumina NovaSeq platform, and 250 bp paired‐end reads were generated.

We used a standard pipeline for 16S rDNA data analysis.^26^ First, raw paired‐end reads were assigned to samples based on their unique barcode sequences. Then, paired‐end reads were merged to obtain raw tags based on 3′ overlapping regions, and barcode and primers were removed. Quality filtering on raw tags was performed, which would keep read error rates < 1%. All of these steps can be completed using VSEARCH, an open‐source and free‐of‐charge multithreaded 64‐bit tool for processing and preparing amplicon analysis. To select representative sequences as proxies of a species, denoising was done by the unoise3 command, which is an implementation of the UNOISE algorithm. After dereplication, denoised sequences were generated and referred to as amplicon sequence variants (ASV). Finally, reference‐based chimera detection was conducted using the Ribosomal Database Project (RDP) as a reference database, and then an ASV table was generated by quantifying normalized ASV counts in each sample. ASV were assigned taxonomy at the genus level with a minimum confidence threshold of 0.1, providing a dimensionality reduction perspective on microbiota.^21^ After removing plastid and non‐bacteria, microbial relative abundance at the genus level was obtained.

### 2.5 Plasma metabolomics analysis

Plasma metabolome was analyzed using the ultrahigh‐performance liquid chromatography coupled with tandem mass spectrometry (UHPLC‐MS/MS) system. Detailed parameters for the nontargeted MS/MS analysis are provided in the *Methods* section of the Supporting Information. A pooled quality control (QC) sample and instrument blanks were used to assess reproducibility and to filter out chemical background contamination. The high degree of overlap in the QC total ion chromatograms (Figure S1) confirmed excellent data consistency and instrument stability. Raw data files were processed using MS‐DIAL (version 4.92) with the following parameters: mass tolerance of 0.01 Da for MS1 and 0.025 Da for MS2; minimum peak height of 50,000 amplitude units; identification score cut‐off of 70%; retention time tolerance of 0.2 min, and MS1 tolerance of 0.01 Da for peak alignment. Adduct ions included [M+H]+, [M+NH_4_]+, [M+Na]+, [M+K]+, [M+H‐H_2_O]+, [M+2Na‐H]+, and [M+2H]2+ in the positive ion mode, and [M‐H]‐, [M‐H_2_O‐H]‐, [M+Na‐2H]‐, [M+Cl]‐, [M+K‐2H]‐, [M+FA‐H]‐, and [M‐2H]2‐ in the negative ion mode. All other parameters were set to default values. For metabolite identification, we used the integrated spectral library provided by the MS‐DIAL team, which combines MassBank, Global Natural Products Social Molecular Networking (GNPS), and ReSpect. Specifically, we employed the “ESI(+)‐MS/MS from authentic standards” and “ESI(‐)‐MS/MS from authentic standards” collections. Features with matching MS/MS spectra according to the above parameters were initially retained. To further reduce false‐positive identifications and ensure data quality, the following filtering steps were applied. The average QC to blank peak area ratio was calculated, and metabolites with a QC/blank peak area ratio < 3 were removed.^27^ Signal reproducibility was tested by calculating the relative standard deviation (RSD) of QC sample technical replicates, and metabolites with RSD > 30% were discarded.^28^ Metabolites with nonzero measurement in at least 80% of samples were included.^29^ Missing values were replaced by 1/5 of the minimum positive values of their corresponding variables. Finally, quantification values of metabolites were normalized by QC samples, made more normally distributed with a generalized log transformation, and standardized using *z*‐scores.^21^

### 2.6 Collection of MRI data

MRI data were collected using a 3.0‐Tesla MR system (Discovery MR750w, General Electric, Milwaukee, WI, USA) with a 24‐channel head coil. During scanning, tight but comfortable foam and earplugs were used to minimize head movement and scanner noise. All participants were instructed to relax, keep their eyes closed but not fall asleep, think of nothing in particular, and move as little as possible. High‐resolution 3D T1‐weighted structural images were acquired using a brain volume (BRAVO) sequence with the following parameters: repetition time (TR) = 8.5 ms; echo time (TE) = 3.2 ms; inversion time (TI) = 450 ms; flip angle (FA) = 12 °; field of view (FOV) = 256 × 256 mm; matrix size = 256 × 256; slice thickness = 1 mm, no gap; 188 sagittal slices; and acquisition time = 296 s. Diffusion tensor imaging (DTI) data were acquired by a spin‐echo single‐shot echo planar imaging (SE‐SS‐EPI) sequence with the following parameters: TR = 10,000 ms; TE = 74 ms; FA = 90 °; FOV = 256 × 256 mm; matrix = 128 × 128; slice thickness = 3 mm without gap; 50 axial slices; 64 diffusion gradient directions (*b* = 1000 s/mm^2^) plus five *b* = 0 reference images; and acquisition time = 700 s. FLAIR data were acquired with the following parameters: TR = 9000 ms; TE = 120.5 ms; TI = 2471 ms; FA = 160 °; FOV = 225 × 225 mm; matrix size = 512 × 512; slice thickness = 6 mm, slice gap = 1 mm; 19 axial slices; and acquisition time = 117 s. Routine T2‐weighted imaging data were acquired with the following parameters: TR = 5057 ms; TE = 127.7 ms; FA = 111 °; FOV = 240 ×240 mm; matrix size = 512 ×512; slice thickness = 5 mm, slice gap = 1.5 mm; 19 axial slices; and acquisition time = 58 s. Resting‐state BOLD functional MRI (fMRI) data were acquired using a gradient‐echo single‐shot echo planar imaging (GRE‐SS‐EPI) sequence with the following parameters: TR = 2000 ms; TE = 30 ms; FA = 90 °; FOV = 220 × 220 mm; matrix size = 64 × 64; slice thickness = 3 mm, slice gap = 1 mm; 35 interleaved axial slices; 185 volumes; and acquisition time = 370 s. All images were visually inspected to ensure that only images without visible artifacts were included in subsequent analyses.

### 2.7 WMH assessment

WMH were graded according to the semiquantitative Fazekas scale,^30^ as periventricular (0 = absence, 1 = caps or pencil‐thin lining, 2 = smooth halo, 3 = irregular WMH extending into deep white matter) and deep (0 = absence, 1 = punctate foci, 2 = beginning confluence of foci, 3 = large confluent areas). Two neurologists independently and blindly scored WMH using T1‐weighted, T2‐weighted, and FLAIR images. Any discrepancies were adjudicated by senior neuroradiologists. The total WMH score (range 0–6) was calculated by summing periventricular and deep subscores, with higher scores indicating greater lesion burden. Additionally, WMH volumes were segmented with Freesurfer's SAMSEG. Linearly co‐registered T1‐weighted and FLAIR images were used as inputs for SAMSEG, and a 0.1 probability threshold was applied to tissue segmentation.^31^ Reassuringly, the semiquantitative visual WMH scores based on the Fazekas scale showed a strong positive correlation with the automated WMH volumes derived from SAMSEG (Spearman *r* = 0.85, *p* < 0.001), supporting the validity and reliability of visual rating in assessing WMH burden.

### 2.8 MRI data preprocessing

T1‐weighted images were preprocessed and parcellated using FreeSurfer (v7.4.1). Key preprocessing steps included motion correction, nonparametric nonuniform intensity normalization, Talairach transform computation, intensity normalization, and skull stripping. For diffusion MRI data, preprocessing was performed using MRtrix3 (v3.0.4) for artifact correction, including Marchenko–Pastur principal component analysis (MP‐PCA) denoising and Gibbs unringing. Subsequently, FSL (v6.0.7) was applied to correct eddy current‐induced distortions and subject movements in diffusion data. Regarding resting‐state fMRI data, preprocessing was conducted using Statistical Parametric Mapping (SPM12) and Data Processing & Analysis for Brain Imaging (DPABI, v5.5). All participants’ BOLD data were within the defined motion thresholds (i.e., translational or rotational motion parameters less than 2 mm or 2 °). Raw fMRI data sequentially underwent dropping of the first 10 volumes and slice‐timing correction. To measure BOLD‐CSF coupling, fMRI data used for BOLD and CSF inflow analyses underwent different preprocessing procedures. For BOLD analysis, fMRI data underwent motion correction, band‐pass filtering (0.01–0.1 Hz), co‐registration of each fMRI volume to the corresponding T1‐weighted structural MRI and then to the Montreal Neurological Institute (MNI) space, spatial Gaussian kernel smoothing (full‐width at half‐maximum [FWHM], 6 mm), and linear and quadratic detrending. For CSF inflow analysis, fMRI data underwent band‐pass filtering (0.01–0.1 Hz) and linear and quadratic detrending, but did not include motion correction, because this step corrupts the voxel slice position information needed for CSF inflow analysis^32^.

### 2.9 CPV estimation

CPV estimation was carried out by using FreeSurfer default cortical and subcortical parcellations based on the Desikan‐Killiany Atlas for bilateral choroid plexus from 3D T1‐weighted images. All the automatic segmentation results underwent thorough inspection and approval by one neuroradiologist. To minimize inter‐subject variations, CPV fraction (CPVF) was calculated as the ratio of CPV to intracranial volume (ICV), as recommended in previous studies^6^.

### 2.10 PVS segmentation

For PVS quantification, we focused on the basal ganglia (BG) as it is evident that PVS in deep white matter predominantly reflect cerebral amyloid‐β pathology characteristic of AD and cerebral amyloid angiopathy,^33^ while PVS in the BG (PVS‐BG) are primarily linked to arteriolosclerosis, a key pathological feature of CSVD.^34^ PVS‐BG segmentation was performed on the parcellations in the native space previously derived from FreeSurfer via an automated quantification pipeline.^35^ Briefly, an adaptive nonlocal mean filtering approach was employed for T1‐weighted images to eliminate bias intensity induced by the Rician noise in MRI data. PVS voxels were retained by applying a filter exclusively to high‐frequency spatial noise and by utilizing a filtering patch with a radius of 1 voxel, which enables maintenance of signal intensities that are spatially repetitive.^36^ Subsequently, the Frangi filter with the default, recommended parameters of α, β, and c^37^ implemented in the Quantitative Imaging Toolkit was applied to denoised T1‐weighted images for estimating a probability‐like measure of “vesselness”. To maximize vessel detection, the scale was set to a broad range of 0.1 to 5 voxels, as suggested in the original paper.^37^ Finally, we utilized a previously optimized threshold of 0.00002 to create a vessel map with the aim of obtaining a binary mask of PVS.^35^ From this mask, PVS‐BG was extracted. To account for interindividual differences in brain size, PVS‐BG volume fraction (PVSVF‐BG), defined as the ratio of PVS‐BG volume to ICV, was calculated.

### 2.11 FW calculation

A single‐shell FW estimation model was performed using the Dipy (v1.10.0) package in Python. In brief, signal within each voxel was fit based on a two‐compartment model, yielding a FW map (representing an isotropic compartment with a fixed diffusion coefficient corresponding to that of water at 37°C) and a FW‐corrected diffusion tensor map. The FW map reflects the relative proportion of FW within each voxel, with values ranging from 0 to 1. Subsequently, the mean FW fraction within the white matter (FW‐WM), excluding regions corresponding to PVS, was derived from the FW map.

### 2.12 ALPS index measurement

ALPS index was calculated using a highly reliable pipeline developed and validated by Liu et al.^38^. First, with preprocessed diffusion MRI data, we calculated fractional anisotropy (FA) and directional diffusivity maps (*x*‐, *y*‐, *z*‐axes) of each participant using the “dtifit” function within FSL. Second, each participant's FA map was co‐registered to the JHU‐ICBM‐FA template, and the transformation matrix was applied to all diffusivity maps by using the FSL command line “flirt”. Next, based on the JHU‐ICBM‐DTI‐81 white matter labeled atlas, projection and association fibers at the level of the lateral ventricle body were identified as the superior corona radiata (SCR) and superior longitudinal fasciculus (SLF), respectively. Spherical regions of interest (ROIs, 5‐mm diameter) were automatically placed in bilateral SCR and SLF regions across all subjects’ diffusivity maps. The ROI centers were defined at the following coordinates in the JHU‐ICBM‐FA template: left SCR (116, 110, 99), left SLF (128, 110, 99), right SCR (64, 110, 99), and right SLF (51, 110, 99). Diffusivity values along the *x*‐, *y*‐, and *z*‐axes (D_xx_, D_yy_, and D_zz_) were extracted from bilateral ROIs for ALPS index calculation. ALPS index was calculated as the ratio of the mean of *x*‐axis diffusivity in the projection area (D_xxproj_) and *x*‐axis diffusivity in the association area (D_xxassoc_) to the mean of *y*‐axis diffusivity in the projection area (D_yyproj_) and *z*‐axis diffusivity in the association area (D_zzassoc_)^39^ as follows: ALPS index = mean (D_xxproj_, D_xxassoc_)/mean (D_yyproj_, D_zzassoc_). Since we had no a priori hypotheses regarding laterality, the average of the left and right ALPS indices was used for subsequent analyses. Recent research has suggested that the ALPS index may also reflect white matter microstructural asymmetry and other physiological diffusion components, rather than serving as a purely specific marker of glymphatic function.^40^ In the present study, we did not apply specific corrections to control for these factors due to the fact that our study sample consisted of individuals with WMH, in whom white matter microstructural alterations are intrinsic to the disease pathology.

### 2.13 BOLD‐CSF coupling quantification

Both global BOLD (gBOLD) and voxel‐wise BOLD (vBOLD) signals were obtained from gray matter regions of the cerebrum as defined by the Harvard–Oxford cortical and subcortical structural atlases, consistent with the previous study.^41^ vBOLD signal was extracted from each voxel in the gray matter mask, and gBOLD signal was computed by averaging the BOLD signals across all gray matter voxels in the MNI space.^11^ CSF inflow signal was extracted by manually segmenting the CSF mask on the bottom slice of the fMRI images and then averaging the fMRI signals within this CSF mask in the original individual space (without spatial registration to the MNI space to avoid spatial blurring from the registration process on such a small region).^11^, ^41^ For gBOLD‐CSF coupling, the cross‐correlation function was computed between gBOLD and CSF signals across different time lags ranging from −10 to 10 s for each participant. The group‐level negative correlation coefficients peaked at the lag of +4 s (Figure S2A), suggesting that the interplay between global hemodynamics and CSF dynamics was strongest at this time point. This time lag was thus selected to compute the strength of gBOLD‐CSF coupling for each participant.^32^ Moreover, we also calculated the cross‐correlation function between the negative derivative of the gBOLD signal and the CSF inflow signal to ensure that CSF signal matched the negative derivative of BOLD oscillation when setting negative value to zero (Figure S2B).^32^ For vBOLD‐CSF coupling, the time lag of a given voxel in a participant was determined by the vBOLD‐CSF cross‐correlation function at its negative peak, and then the vBOLD‐CSF coupling strength was computed at this lag, similar to gBOLD‐CSF coupling. This procedure was repeated for each voxel, yielding a vBOLD‐CSF coupling map along with a time lag map per participant. To ease analysis, vBOLD‐CSF coupling values were extracted from eight well‐established canonical brain networks. The 7 cortical networks were defined as the visual, somatomotor, dorsal attention, ventral attention, limbic, frontoparietal, and default networks according to the Yeo et al. study.^42^ The Human Brainnetome Atlas was adopted to define the subcortical network, including the amygdala, hippocampus, basal ganglia, and thalamus.

### 2.14 Statistical analyses

Statistical analyses were carried out using SPSS 23.0 (SPSS, Chicago, IL, United States) and R (v4.4.1). To compare demographic, clinical, and cognitive characteristics across HC, WMH‐NC, and WMH‐CI, we employed one‐way analysis of variance (ANOVA) for continuous variables and chi‐square test for categorical variables. Overall group differences in gut microbiome and plasma metabolome were initially examined using a combination of principal coordinate analysis (PCoA) based on Bray‐Curtis dissimilarity and permutational multivariate analysis of variance (PERMANOVA; 1000 permutations). Next, gut microbiome, plasma metabolome, CPVF, PVSVF‐BG, FW‐WM, and BOLD‐CSF coupling were compared across the three groups using the Kruskal–Wallis test, while the ALPS index was compared using one‐way ANOVA. These analyses were adjusted for potential confounders, including age, sex, education, and vascular risk factors. Notably, when analyzing gut microbiome and plasma metabolome, we excluded participants who had received antibiotics within the past 3 months (four HC, three WMH‐NC, and three WMH‐CI).

To investigate the microbiota‐metabolites‐glymphatic function‐cognition relationship, a comprehensive analytical framework was implemented. Initially, partial Spearman's correlation analyses were conducted in all participants between variables of microbiota, metabolites, glymphatic function, and cognition, which exhibited significant group differences. More importantly, to test whether the association between microbiota and cognition was mediated by metabolites and/or glymphatic function, mediation analysis was performed using the PROCESS macro, with microbiota (X) and cognition (Y) as independent and dependent variables, and metabolites (M1) and glymphatic function measures (M2) as mediating variables. In the mediation model, we sought to establish three pathways: (1) X → M1 → M2 → Y; (2) X → M1 → Y; and (3) X → M2 → Y. All pathways were reported as unstandardized ordinary least squares regression coefficients, namely, total effect of X on Y (c) = indirect effect of X on Y through M1 (a1 × b1) + indirect effect of X on Y through M2 (a2 × b2) + indirect effect of X on Y through M1 and M2 (a1 × d × b2) + direct effect of X on Y (c’). Age, sex, education, and vascular risk factors were considered nuisance variables. The significance analysis was based on 5,000 bootstrap realizations, and a significant indirect effect is indicated when the bootstrap 95% confidence interval does not include zero.

## 3 RESULTS

### 3.1 Demographic, clinical, and cognitive characteristics

This study enrolled 145 participants, comprising 56 HC, 40 WMH‐NC, and 49 WMH‐CI. Demographic, clinical, and cognitive characteristics are presented in Table 1. There were no significant differences in age, sex, or education among the three groups. Regarding vascular risk factors, the prevalence of diabetes, dyslipidemia, and smoking was comparable across the three groups, while hypertension was more prevalent in WMH‐NC and WMH‐CI than in HC. With respect to cognition, WMH‐CI showed significantly lower scores in MoCA, MMSE, AVLT total, FDS, and BDS compared to HC and WMH‐NC.

**TABLE 1:** Demographic, clinical, and cognitive characteristics of the participants

Characteristic	HC (*n* = 56)	WMH‐NC (*n* = 40)	WMH‐CI (*n* = 49)	Statistical test	Post hoc analysis
Age, y, mean (SD)	61.50 (7.52)	63.75 (6.84)	64.94 (7.88)	*F* = 2.879, *p* = 0.059	HC = WMH‐NC = WMH‐CI
Female, *n*(%)	30 (53.57)	14 (35.00)	24 (48.98)	*χ* ^2^ = 3.360, *p* = 0.186	HC = WMH‐NC = WMH‐CI
Education, *y*, mean (SD)	9.02 (4.33)	7.91 (4.73)	7.94 (3.85)	*F* = 1.111, *p* = 0.332	HC = WMH‐NC = WMH‐CI
Vascular risk factors					
Hypertension, *n* (%)	12 (21.43)	26 (65.00)	26 (53.06)	*χ* ^2^ = 20.356, *p* < 0.001	HC < WMH‐NC = WMH‐CI
Diabetes, *n* (%)	3 (5.36)	7 (17.50)	9 (18.37)	*χ* ^2^ = 4.823, *p* = 0.090	HC = WMH‐NC = WMH‐CI
Dyslipidemia, *n* (%)	11 (19.64)	6 (15.00)	6 (12.24)	*χ* ^2^ = 1.102, *p* = 0.576	HC = WMH‐NC = WMH‐CI
Smoking, *n* (%)	13 (23.21)	12 (30.00)	9 (18.37)	*χ* ^2^ = 1.663, *p* = 0.435	HC = WMH‐NC = WMH‐CI
Cognition					
MoCA, mean (SD)	26.00 (2.37)	24.35 (3.30)	17.20 (5.20)	*F* = 76.705, *p* < 0.001	HC = WMH‐NC > WMH‐CI
MMSE, mean (SD)	28.70 (1.26)	27.68 (2.02)	22.53 (5.09)	*F* = 52.138, *p* < 0.001	HC = WMH‐NC > WMH‐CI
AVLT total^a^, mean (SD)	50.11 (9.38)	45.55 (11.15)	34.85 (11.81)	*F* = 26.584, *p* < 0.001	HC = WMH‐NC > WMH‐CI
FDS^b^, mean (SD)	13.09 (3.48)	12.41 (2.78)	10.22 (2.99)	*F* = 11.373, *p* < 0.001	HC = WMH‐NC > WMH‐CI
BDS^b^, mean (SD)	7.20 (2.05)	6.77 (2.71)	4.77 (1.98)	*F* = 16.630, *p* < 0.001	HC = WMH‐NC > WMH‐CI

Abbreviations: AVLT, Auditory Verbal Learning Test; BDS, Backward Digit Span; FDS, Forward Digit Span; HC, healthy controls; MMSE, Mini‐Mental State Examination; MoCA, Montreal Cognitive Assessment; SD, standard deviation; WMH‐CI, white matter hyperintensities with cognitive impairment; and WMH‐NC, white matter hyperintensities with normal cognition.

^a^ The data are available for 47 from 49 WMH‐CI.

^b^ The data are available for 55 from 56 HC, 39 from 40 WMH‐NC, and 48 from 49 WMH‐CI.

### 3.2 Group differences in gut microbiome

**FIGURE 2:**
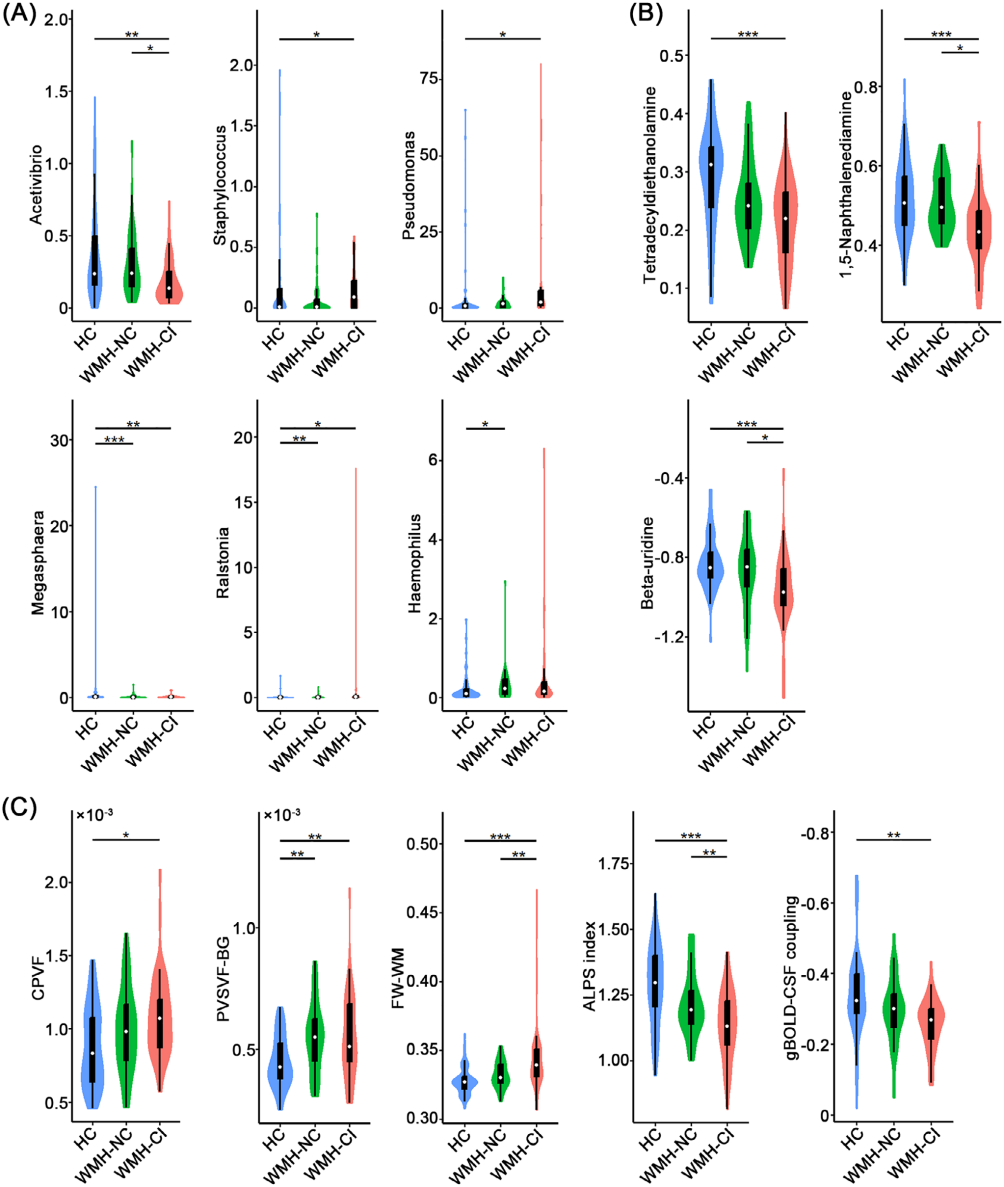
Differences in gut microbiome, plasma metabolome, and glymphatic function across HC, WMH‐NC, and WMH‐CI. (A) Violin plots of the relative abundance of the microbial taxa showing significant group differences at the genus level. (B) Violin plots of quantification values of the plasma metabolites showing significant group differences. (C) Violin plots of the glymphatic function measures showing significant group differences. ^*^ *p* < 0.05, ^**^ *p* < 0.01, ^***^ *p* < 0.001. ALPS index, index of diffusivity along the perivascular spaces; CPVF, choroid plexus volume fraction; FW‐WM, free water fraction within the white matter; gBOLD‐CSF coupling, coupling between global blood oxygen level‐dependent and cerebrospinal fluid signals; HC, healthy controls; PVSVF‐BG, volume fraction of perivascular spaces in the basal ganglia; WMH‐CI, white matter hyperintensities with cognitive impairment; WMH‐NC, white matter hyperintensities with normal cognition.

Significant overall differences in microbial community composition among the three groups were detected by PERMANOVA (*p* = 2.50 × 10^−2^) (Figure S3). Kruskal–Wallis test revealed significant differences in relative abundance of 11 microbial taxa at the genus level among the three groups (*p *< 0.05) (Table S1). Post hoc Dunn's tests, with Bonferroni correction for three pairwise comparisons within each taxon, confirmed statistically robust group differences for six microbial taxa: *Acetivibrio, Staphylococcus, Pseudomonas, Megasphaera, Ralstonia*, and *Haemophilus* (Figure 2A). These taxa exhibited three major alteration patterns: (1) WMH‐CI‐specific reduction: *Acetivibrio* was lower in WMH‐CI than in both HC and WMH‐NC; (2) progressive increase from HC to WMH‐CI: *Staphylococcus* and *Pseudomonas* elevated in WMH‐CI relative to HC; and (3) WMH‐related alterations: *Megasphaera* decreased, and *Ralstonia* increased in both WMH groups compared with HC. A modest elevation of *Haemophilus* was also noted in WMH‐NC relative to HC. Detailed statistics are provided in Table S1.

### 3.3 Group differences in plasma metabolome

After quality control, data filtering, and normalization, 144 and 141 metabolites were obtained in positive and negative ion modes, respectively. Significant overall differences in metabolomic profiles among the three groups were observed in both positive and negative ion modes (PERMANOVA‐*p* = 1.00 × 10^−3^ for both modes) (Figure S4). Kruskal–Wallis test revealed significant differences in tetradecyldiethanolamine, 1,5‐naphthalenediamine, and beta‐uridine among the three groups in the positive ion mode (*p* < 0.05, Bonferroni corrected) (Figure 2B and Table S2), but no significant group differences in the negative ion mode (Table S3). These metabolites exhibited two major alteration patterns: (1) progressive decrease from HC to WMH‐CI: tetradecyldiethanolamine decreased in WMH‐CI relative to HC; (2) WMH‐CI‐specific reduction: 1,5‐naphthalenediamine and beta‐uridine were lower in WMH‐CI than in both HC and WMH‐NC. Detailed statistics are provided in Table S2. For completeness, we also analyzed the unidentified metabolic features and found that 11 features in the positive ion mode (Table S4) and 5 features in the negative ion mode (Table S5) showed significant differences among the three groups.

### 3.4 Group differences in glymphatic function

Kruskal–Wallis test revealed significant differences in CPVF, PVSVF‐BG, FW‐WM, and BOLD‐CSF coupling among the three groups, and one‐way ANOVA demonstrated a significant group difference in ALPS index (*p* < 0.05) (Figure 2C). Post hoc pairwise comparisons with Bonferroni correction revealed three major patterns: (1) progressive alterations from HC to WMH‐CI: CPVF larger and gBOLD‐CSF coupling weaker in WMH‐CI than in HC; (2) WMH‐related increase: PVSVF‐BG elevated in both WMH‐NC and WMH‐CI compared with HC; (3) WMH‐CI specific alterations: FW‐WM elevated and ALPS index reduced in WMH‐CI relative to both HC and WMH‐NC. Detailed statistics are provided in Table S6. At the network level, vBOLD‐CSF coupling in the dorsal attention, ventral attention, frontoparietal, and subcortical networks showed the same pattern of change as gBOLD‐CSF coupling (Figure S5), while no significant group differences were observed for the visual, somatomotor, limbic, or default networks.

### 3.5 Associations between gut microbiota, plasma metabolites, glymphatic function, and cognition

**FIGURE 3:**
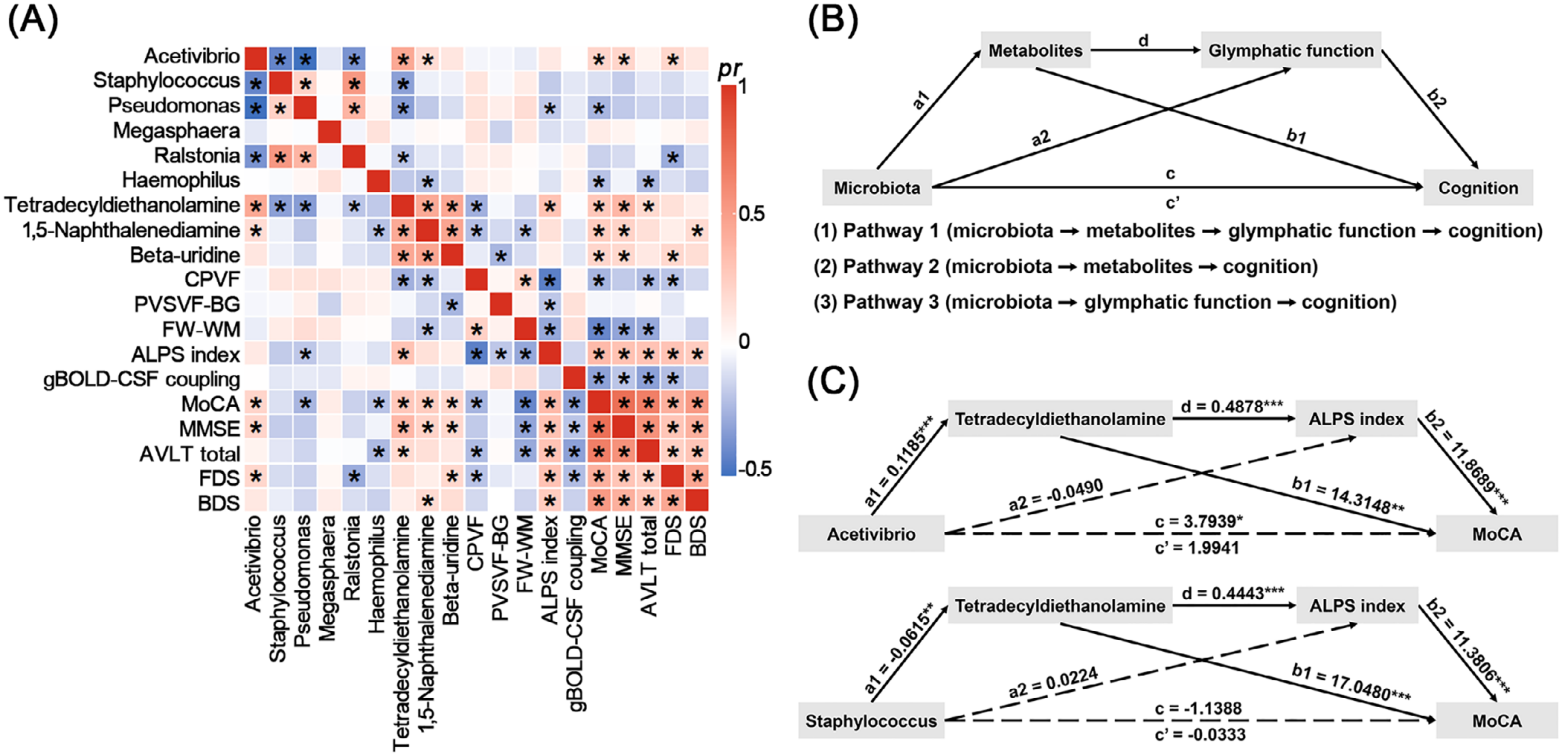
Associations between gut microbiota, plasma metabolites, glymphatic function, and cognition. (A) Heatmap of the correlations between gut microbiota, plasma metabolites, glymphatic function measures, and cognition in all participants. (B) Conceptual mediation pathways linking microbiota, metabolites, glymphatic function, and cognition. (C) Representative mediation results. ^*^ *p* < 0.05, ^**^ *p* < 0.01, ^***^ *p* < 0.001. ALPS index, index of diffusivity along the perivascular spaces; AVLT, auditory verbal learning test; BDS, Backward Digit Span; CPVF, choroid plexus volume fraction; FDS, Forward Digit Span; FW‐WM, free water fraction within the white matter; gBOLD‐CSF coupling, coupling between global blood oxygen level‐dependent and cerebrospinal fluid signals; MMSE, Mini‐Mental State Examination; MoCA, Montreal Cognitive Assessment; PVSVF‐BG, volume fraction of perivascular spaces in the basal ganglia.

Partial Spearman's correlation analyses revealed a range of associations between gut microbiota, plasma metabolites, glymphatic function measures, and cognition in all participants (Figure 3A). As illustrated in the conceptual mediation model (Figure 3B), we further explored three possible pathways linking microbiota to cognition via metabolites and/or glymphatic function. Representative mediation results are shown in Figure 3C. Specifically, the positive association between *Acetivibrio* and MoCA was significantly mediated by tetradecyldiethanolamine and ALPS index (indirect effect = 0.6858, 95% CI: 0.2041, 1.5237) as well as by tetradecyldiethanolamine alone (indirect effect = 1.6957, 95% CI: 0.2817, 3.4571). Likewise, the negative association between *Staphylococcus* and MoCA was mediated by tetradecyldiethanolamine and ALPS index (indirect effect = −0.3111, 95% CI: −1.0113, −0.0604) as well as by tetradecyldiethanolamine alone (indirect effect = −1.0490, 95% CI: −3.4158, −0.1760). Detailed mediation results are provided in Table S7.

## 4 DISCUSSION

By using a combination of 16S rDNA amplicon sequencing, untargeted metabolomics, and multi‐modal MRI, we comprehensively investigated the microbiota‐gut‐brain mechanism underlying cognitive heterogeneity in WMH. Gut microbiomics analysis revealed significant differences in six bacterial genera across HC, WMH‐NC, and WMH‐CI. Plasma metabolomics analysis identified three metabolites showing significant differences across the three groups. Multi‐modal MRI analysis demonstrated significant group differences in all glymphatic function markers. Integration of these multi‐omics changes uncovered multiple associations among microbiota, metabolites, glymphatic function measures, and cognition. More importantly, mediation analysis supported two potential pathways linking microbiota to cognition. These findings provide preliminary insights into a biological framework that may help explain vulnerability and/or resilience to WMH‐CI, potentially informing more targeted interventions to preserve cognition and prevent VCI.

Gut microbiomics analysis identified six bacterial genera with distinct change patterns across the three groups, potentially reflecting progressive microbiota‐gut‐brain axis dysregulation from HC to WMH‐NC and ultimately to WMH‐CI. *Acetivibrio* was significantly depleted in WMH‐CI, suggesting that its reduction is specifically associated with WMH‐CI rather than WMH in general. This highlights *Acetivibrio* as a potential biomarker of cognitive vulnerability in WMH. *Acetivibrio* ferments carbohydrates to produce acetic acid,^43^ the most common short‐chain fatty acid (SCFA). SCFAs modulate microglial activation, blood–brain barrier integrity, and neuroinflammation, which are implicated in various neuropsychiatric disorders.^44^, ^45^, ^46^, ^47^, ^48^, ^49^ The depletion of *Acetivibrio* in WMH‐CI may disrupt gut‐brain anti‐inflammatory and metabolic balance, potentially contributing to cognitive vulnerability. *Staphylococcus* and *Pseudomonas* were enriched in WMH‐CI relative to HC, implying the importance of their abundance increases in WMH progression and cognitive deterioration. Intriguingly, both *Staphylococcus* and *Pseudomonas* are well‐known opportunistic pathogens. *Staphylococcus*‐induced peripheral inflammation or neuroinflammation is involved in the pathogenesis of mental disorders.^50^, ^51^ In parallel, *Pseudomonas* aeruginosa infection can promote neuronal tauopathy and Aβ amyloidosis,^51^, ^52^ key features of neurodegenerative diseases and major contributors to cognitive decline. The enrichment of these bacteria may indicate a shift toward a more pro‐inflammatory and neurodegeneration‐prone microbial environment, resulting in WMH pathology and cognitive decline. Both WMH‐NC and WMH‐CI demonstrated reduced *Megasphaera* and elevated *Ralstonia* relative to HC, suggesting that these changes may relate to cerebrovascular pathology regardless of cognitive status. *Megasphaera*, a key lactate‐degrading bacterium, is regarded as beneficial.^53^ Stroke‐model evidence demonstrated that restoring *Megasphaera* helped preserve brain‐gut barrier integrity.^54^ In contrast, the enrichment of *Ralstonia*, an aerobic gram‐negative opportunistic pathogen,^55^ represents a potentially detrimental microbial shift. This finding echoes reports in neurological disorders, including autism.^56^ Notably, *Haemophilus* showed a unique pattern, being significantly elevated in WMH‐NC, but not in WMH‐CI. As a potentially pathogenic genus,^57^ this transient increase may reflect early‐stage gut dysbiosis. Microbial changes are not always unidirectional. Certain harmful taxa may temporarily expand in intermediate stages and later be suppressed or replaced as the disease environment evolves. Collectively, the distinct change patterns of these six bacterial genera highlight the complexity of gut microbiota dysbiosis in WMH. The depletion of beneficial bacteria may create ecological niches that facilitate the expansion of opportunistic pathogens, disrupting gut‐brain axis homeostasis through inflammation, metabolic dysregulation, and neurotransmitter imbalance. Understanding these intricate relationships is crucial for developing microbiota‐based interventions to prevent or treat WMH‐related CI.

Plasma metabolomics analysis demonstrated significant differences in tetradecyldiethanolamine, 1,5‐naphthalenediamine, and beta‐uridine across groups. These showed two distinct alteration patterns, with one reflecting a progressive shift from HC to WMH‐CI and the other indicating a WMH‐CI‐specific effect. Tetradecyldiethanolamine showed a stepwise reduction across groups. As a lipid‐soluble antimicrobial compound,^58^ its reduction may reflect impaired membrane stability or disrupted gut microbial balance during disease progression. This gradual decline indicates a possible role in maintaining brain or gut barrier integrity, the disruption of which may contribute to WMH pathology and cognitive decline. Nevertheless, 1,5‐naphthalenediamine and beta‐uridine showed the WMH‐CI specific alteration pattern. 1,5‐Naphthalenediamine is an environmental or occupational compound with unclassifiable carcinogenicity.^59^, ^60^, ^61^ Surprisingly, its plasma levels were significantly lower in WMH‐CI. This paradox may stem from redistribution rather than enhanced detoxification: systemic inflammation and blood‐brain barrier dysfunction in WMH‐CI could drive lipophilic compounds into peripheral tissues or brain parenchyma, reducing circulating concentrations. Thus, low plasma levels may still reflect neurotoxic exposure. Beta‐uridine is an endogenous nucleoside fundamentally involved in RNA metabolism and nucleotide biosynthesis.^59^ It also serves as a precursor supporting phosphatidylcholine synthesis, which is essential for neuronal membrane integrity and synaptic function.^62^ Of note, uridine crosses the blood‐brain barrier^63^ and exerts neuroprotective effects by promoting synaptic membrane formation, neuronal repair, and neurotransmission.^64^ Studies have demonstrated its capacity to improve cognitive function.^64^, ^65^ Therefore, reduced uridine in WMH‐CI may reflect impaired neurochemical support, linking its depletion to cognitive decline. In summary, the distinct metabolite alterations highlight the complex interplay between environmental exposures and intrinsic metabolic disruptions in WMH‐CI, potentially guiding novel strategies to mitigate WMH‐related cognitive decline.

Multi‐modal MRI analysis showed significant group differences in glymphatic function with distinct patterns. CPVF progressively increased from HC to WMH‐NC to WMH‐CI, possibly as an early compensatory response to boost CSF production and offset dysfunction.^6^, ^66^ PVSVF‐BG showed a WMH‐related increase pattern. While PVS alterations link to CSVD burden,^13^ PVS‐BG may not strongly associate with cognition^15^ and may precede other CSVD features.^67^, ^68^ These findings align with our observation that PVSVF‐BG increases in WMH regardless of cognition, implying that PVS‐related glymphatic dysfunction arises early in CSVD and reflects vascular pathology rather than cognitive decline. FW‐WM and ALPS index exhibited WMH‐CI‐specific alterations. Elevated FW has been correlated with cognitive decline^14^, ^15^ and causes cerebral structural abnormalities.^14^ Complementing these findings, a declined ALPS index has been independently linked to cognitive impairment.^8^, ^15^, ^39^, ^69^ This supports the hypothesis that impaired CSF‐ISF exchange may lead to waste and inflammatory mediator buildup, triggering neuroinflammation and neuronal damage that contribute to cognitive and neurological impairments.^70^ Finally, gBOLD‐CSF progressively decreased from HC to WMH‐CI. Reduced BOLD‐CSF coupling has been linked to neurodegenerative pathology, cognitive decline, and toxic protein deposition.^17^, ^71^ A similar pattern in the AD continuum supports progressive glymphatic failure as a common neurodegeneration pathway.^72^ Taken together, these findings suggest a sequential glymphatic failure model. The progressive enlargement of CPVF may reflect an early compensatory mechanism for boosting CSF production, while PVSVF‐BG alterations likely represent early vascular responses affecting CSF inflow. FW‐WM and ALPS index alterations point to downstream impairment in CSF‐ISF exchange and ISF outflow, both of which may contribute directly to neuroinflammation and cognitive decline. gBOLD‐CSF coupling further provides an integrative marker of global glymphatic dysfunction across disease stages. This functional dissociation underscores the importance of assessing multiple dimensions of glymphatic activity, which may not only clarify the pathophysiological heterogeneity of CSVD but also provide a framework for stage‐specific risk prediction and therapeutic targeting.

Correlation and mediation analyses uncovered a unified microbiota–metabolites–glymphatic function–cognition axis that may underlie cognitive heterogeneity in WMH. *Acetivibrio* was positively associated with MoCA, mediated by tetradecyldiethanolamine and ALPS index. It is known that *Acetivibrio* produces SCFA that can enhance blood‐brain barrier integrity and reduce neuroinflammation.^43^, ^44^, ^45^, ^46^ Our findings extend these insights by suggesting that *Acetivibrio* may exert its beneficial effects through modulation of specific lipid metabolites like tetradecyldiethanolamine, which may influence cognition directly or via supporting glymphatic function (ALPS index). This aligns with evidence that lipid metabolites influence astrocytic membrane properties^73^, thereby affecting perivascular fluid dynamics and CSF‐ISF exchange.^68^ *Staphylococcus* was negatively associated with MoCA, similarly mediated by tetradecyldiethanolamine and ALPS index. *Staphylococcus* increases gut permeability and systemic inflammation, potentially disrupting phospholipid metabolism and impairing cognition directly or via glymphatic dysfunction.^74^ Together, these findings delineate two mechanistic pathways through which the gut microbiota may influence cognitive outcomes: (1) microbiota → metabolites → glymphatic system → cognition, and (2) microbiota → metabolites → cognition. These findings refine our understanding of how peripheral microbial signals can modulate central neurophysiology in WMH.

This study has several limitations. First, its correlational design precludes causal inference, requiring longitudinal and mechanistic studies to confirm multi‐omics associations. Second, untargeted metabolomics provided only relative metabolite quantification, necessitating targeted analysis for precise measurement. Third, although multi‐modal MRI was used to assess glymphatic function, these measures are indirect and may be influenced by factors beyond glymphatic clearance. Notably, the ALPS index may be influenced by white matter microstructural asymmetry and other physiological diffusion signals.^40^ We did not adjust for these factors in our analysis, which may influence the interpretation of our results. Fourth, while mediation analysis was central to our study for identifying microbiota–cognition pathways, we must acknowledge its limitations. This method requires defining variable relationships based on existing knowledge and assumptions, which may not fully reflect the underlying biological complexity. Finally, we did not control for oral probiotics or yogurt intake. We reasoned that consumers were randomly distributed, making a systematic bias unlikely, and that the high variability in usage patterns made precise quantification impractical.

In conclusion, our integrative multi‐omics study established plausible microbiota–metabolites–glymphatic function–cognition associations that may hold promise to better account for cognitive heterogeneity in WMH. More broadly, these findings may have important clinical implications for developing interventions targeting aspects of the identified pathways to preserve cognitive function and prevent VCI.

## CONFLICT OF INTEREST STATEMENT

The authors declare no conflict of interest. Author disclosures are available in the Supporting Information.

## CONSENT STATEMENT

All participants provided written informed consent after receiving a full explanation of the study procedures, and the protocol was approved by the institutional review board.

## Supporting information



Supporting Information

Supporting Information

Supporting Information
