# Supplementary material for: Resolving cognitive heterogeneity in white matter hyperintensities through integrated analyses of microbiome, metabolome, and brain glymphatic function
Source: Alzheimers Dement. 2026 Feb 17;22(2):e71201. doi: 10.1002/alz.71201 (PMC12910248; doi:10.1002/alz.71201)
Supplement: Supplementary file 2 — Supporting Information [file ALZ-22-e71201-s001.doc]

**Supplementary Materials**

**METHODS**

**UHPLC–MS/MS analysis**

Ultrahigh performance liquid chromatography coupled with tandem mass spectrometry (UHPLC-MS/MS) analysis was performed using a Thermo Fisher UHPLC system (Germany) equipped with a Hypesil Gold column (100 × 2.1 mm, 1.9 µm) and coupled to a Q Exactive™ HF-X mass spectrometer (Thermo Fisher, Germany). Chromatographic conditions: The column temperature was maintained at 40°C with a flow rate of 0.2 mL/min. For positive ion mode, mobile phase A was 0.1% formic acid and mobile phase B was methanol. For negative ion mode, mobile phase A was 5 mM ammonium acetate and mobile phase B was methanol. The solvent gradient was programmed as follows: 2% B at 0-1.5 min; 2-85% B from 1.5-3.0 min; 85-100% B from 3.0-10.0 min; 100-2% B from 10.0-10.1 min. Mass spectrometric conditions: ESI source parameters were set as follows: spray voltage: 3.5 kV; sheath gas flow rate: 35 psi; auxiliary gas flow rate: 10 L/min; ion transfer tube temperature: 320°C; radio frequency lens level: 60; auxiliary gas heater temperature: 350°C. Data were acquired in both positive and negative ionization modes. The *m/z* range was 100-1500. MS/MS spectra were acquired using data-dependent acquisition.

**
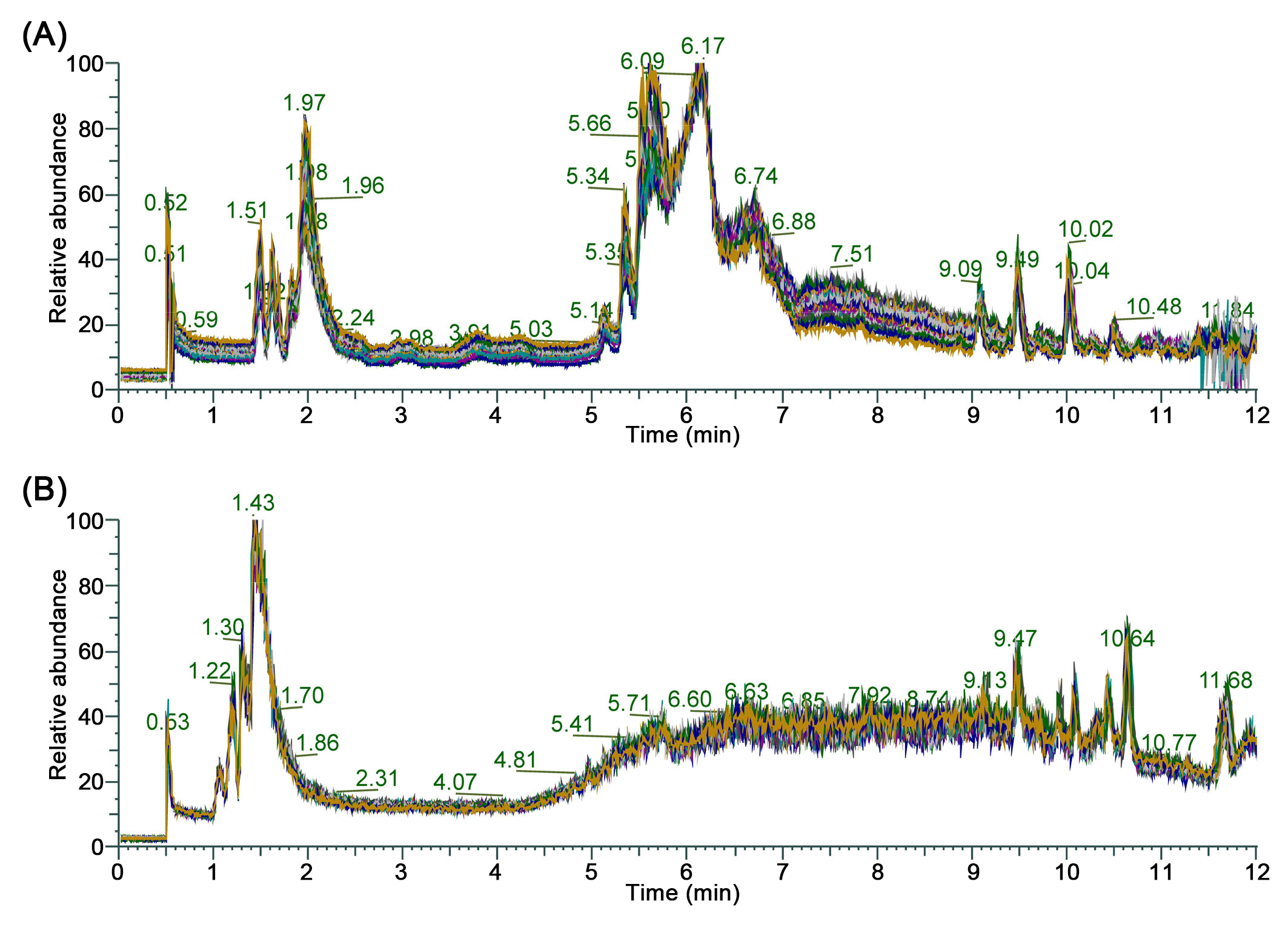
**

**Figure S1.** TIC overlap diagram of QC samples. (A) Positive ion mode. (B) Negative ion mode. Abbreviations: QC, quality control; TIC, total ion chromatograms.

**
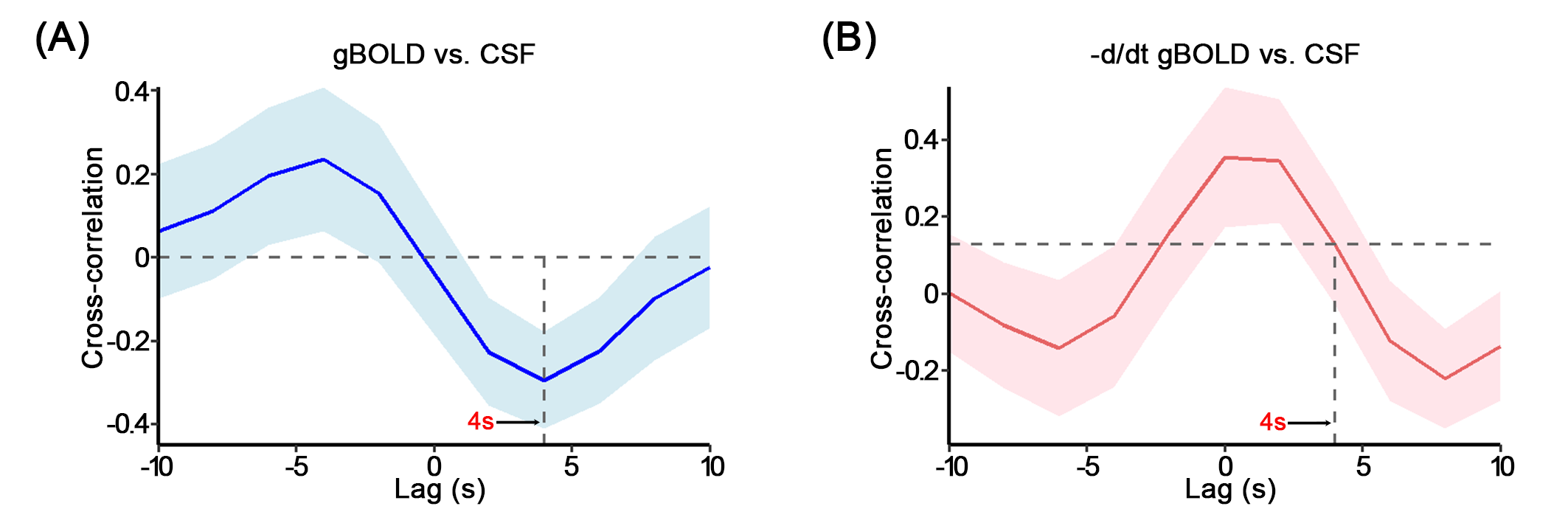
**

**Figure S2.** Cross-correlation analysis between gBOLD and CSF signals. (A) The cross-correlation function between gBOLD and CSF signals averaged across participants (blue line). The blue area indicates the SD, and the vertical dashed line marks the +4 s time lag as the negative peak of the mean cross-correlation (*r* = -0.29, *P* = 0.001, 1,000 times permutation test). (B) The cross-correlation function between the zero-threshold negative derivative of gBOLD signal and CSF signal averaged across participants (pink line). The pink area indicates the SD, and the vertical dashed line indicates the strongest correlation at +4 s. Abbreviations: CSF, cerebrospinal fluid; gBOLD, global blood oxygen level-dependent; SD, standard deviation.


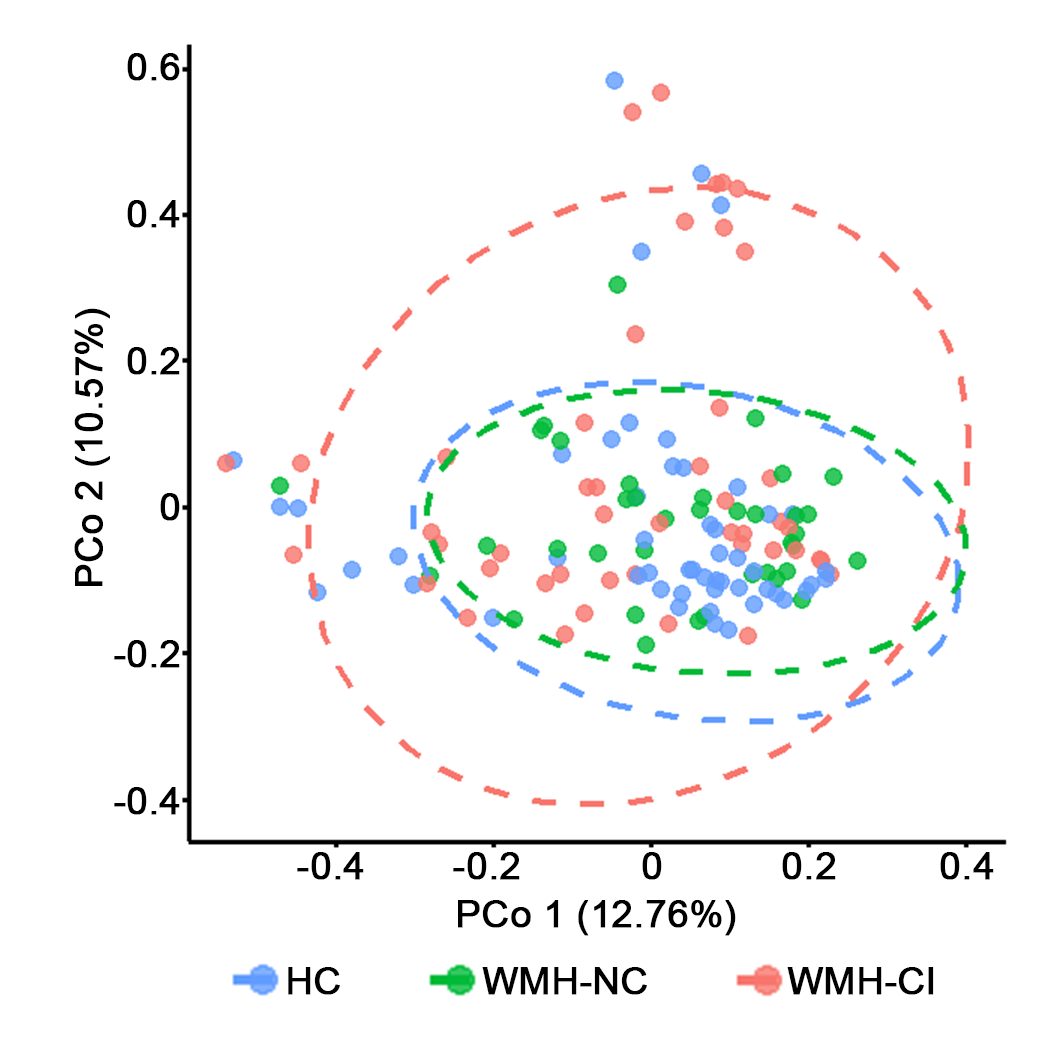


**Figure S3.** PCoA plots of microbial community. Abbreviations: HC, healthy controls; PCoA, principal coordinate analysis; WMH-CI, white matter hyperintensities with cognitive impairment; WMH-NC, white matter hyperintensities with normal cognition.


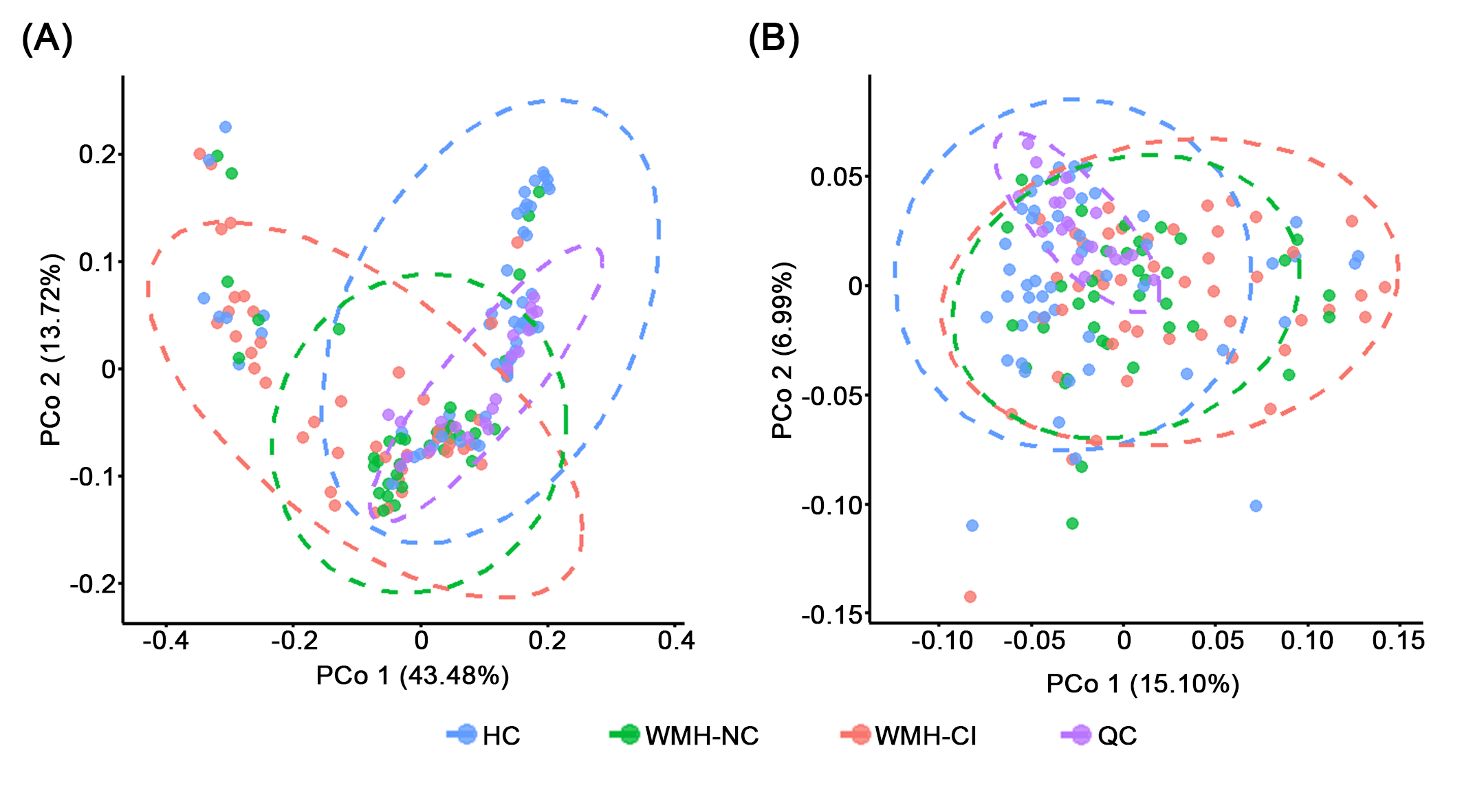


**Figure S4.** PCoA plots of metabolomic profiles. (A) Positive ion mode. (B) Negative ion mode. Abbreviations: HC, healthy controls; PCoA, principal coordinate analysis; QC, quality control; WMH-CI, white matter hyperintensities with cognitive impairment; WMH-NC, white matter hyperintensities with normal cognition.

**
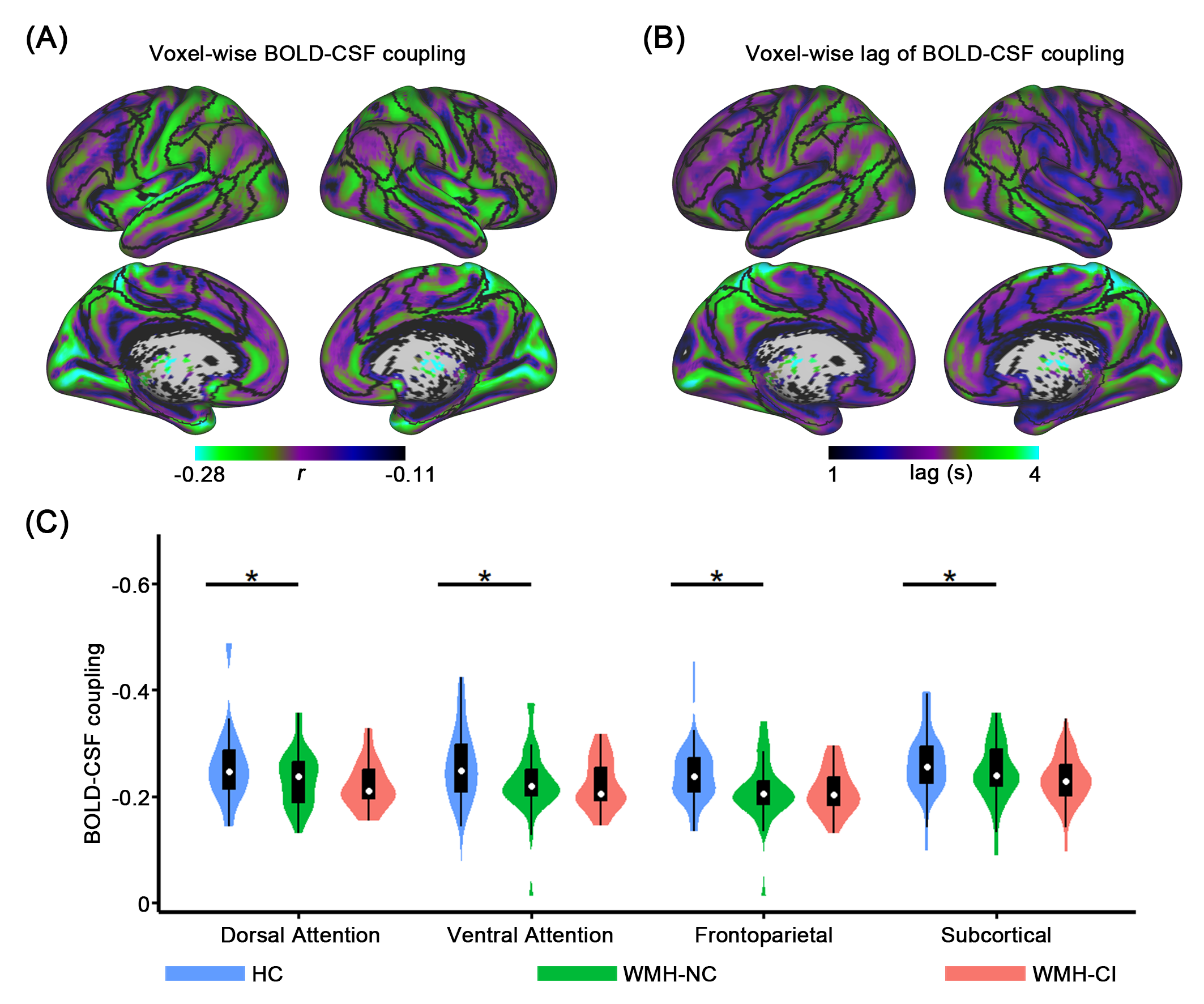
**

**Figure S5.** The coupling strength and time lags between voxel-wise BOLD and CSF signals. (A) Averaged strength map of voxel-wise BOLD-CSF coupling across participants. (B) Averaged time lag map of voxel-wise BOLD-CSF coupling across participants. (C) Canonical networks showing significant group differences in BOLD-CSF coupling. **P* < 0.05, ***P* < 0.01, ****P* < 0.001. Abbreviations: BOLD-CSF coupling, coupling between blood oxygen level-dependent and cerebrospinal fluid signals; HC, healthy controls; WMH-CI, white matter hyperintensities with cognitive impairment; WMH-NC, white matter hyperintensities with normal cognition.
